# Polymorphisms in DNA Repair Genes and Susceptibility to Glioma in a Chinese Population

**DOI:** 10.3390/ijms14023314

**Published:** 2013-02-05

**Authors:** Wei-Ran Pan, Gang Li, Jun-Hong Guan

**Affiliations:** Department of Neurosurgery, Shengjing Affiliated Hospital of China Medical University, Shenyang 110004, China; E-Mails: gangli2003@126.com (G.L.); junhg1984@163.com (J.-H.G.)

**Keywords:** ERCC1, XRCC1, XRCC3, polymorphisms, glioma

## Abstract

The excision repair cross-complementing rodent repair deficiency complementation group 1 (ERCC1), and X-ray repair cross-complementing group 1 (XRCC1) genes appear to protect mammalian cells from the harmful effects of ionizing radiation. We conducted a large case-control study to investigate the association of polymorphisms in ERCC1 C118T, ERCC1 C8092A, XRCC1 A194T, XRCC1 A194T, and XRCC3 C241T, with glioma risk in a Chinese population. Five single nucleotide polymorphisms (SNPs) were genotyped, using the MassARRAY IPLEX platform, in 443 glioma cases and 443 controls. Association analyses based on an χ^2^ test and binary logistic regression were performed to determine the odds ratio (OR) and a 95% confidence interval (95% CI) for each SNP. For XRCC1 Arg194Trp, the variant genotype T/T was strongly associated with a lower risk of glioma cancer when compared with the wild type C/C (OR = 2.45, 95% CI = 1.43–4.45). Individuals carrying the XRCC1 399A allele had an increased risk of glioma (OR = 1.33, 95% CI = 1.02–1.64). The XRCC3 241T/T genotype was associated with a strong increased glioma risk (OR = 3.78, 95% CI = 1.86–9.06). Further analysis of the interactions of two susceptibility-associated SNPs, XRCC1 Arg194Trp and XRCC3 Thr241Met, showed that the combination of the XRCC1 194T and XRCC3 241T alleles brought a large increase in glioma risk (OR = 2.75, 95% CI = 1.54–4.04). XRCC1 Arg194Trp, XRCC1 Arg399Gln, and XRCC3 C241T, appear to be associated with susceptibility to glioma in a Chinese population.

## 1. Introduction

Tumors of the central nervous system (CNS) account for about 2% of all cancers, and its morbidity is about 4.2/10^5^ to 5.4/10^5^ people per year, worldwide [1]. Although the incidence of CNS tumors is small, compared with other cancers, these are among the most serious human malignancies, since they affect coordination and the integration of all organic activities. Moreover, as each region of the brain has a vital function, total surgical removal of the organ or tumor, which is used with other cancers, cannot be applied to cure brain tumors [2]. Gliomas are the most common tumors of the CNS. Despite the remarkable progress in the characterization of the molecular pathogenesis of gliomas, these tumors remain incurable and, in most cases, resistant to treatment due to their molecular heterogeneity.

Although many studies have been conducted on the etiology of glioma, it is still not completely understood. The only established environmental risk factors are ionizing radiation and ultraviolet rays, and the low exposure to these types of radiation, in daily life, may explain the low incidence rate of this cancer [3,4]. Both types of radiation cause an accumulation of DNA damage, including oxidative DNA damage, single- and double-strand breaks in DNA chains, and DNA–DNA or DNA–protein cross-links. This DNA damage may lead to tumor development and various cellular dysfunctions [5]. Several complex systems of DNA repair work against DNA damage and prevent mutagenesis, including base excision repair (BER), nucleotide excision repair (NER), double-strand break repair (SSBR), and homologous recombination repair (HRR). BER, NER, and HRR, constitute the main defenses against lesions generated by ionizing radiation, alkylating agents, and reactive oxygen species [6]. The excision repair cross-complementing rodent repair deficiency complementation group 1 (ERCC1) gene is reported to be a crucial gene in the NER pathway, and ERCC1 polymorphisms can modify the function of NER and, thus, influence the risk of human cancers. X-ray repair cross-complementing groups 1 (XRCC1) polymorphisms facilitate the BER and SSBR processes [7–9]. X-ray repair cross-complementing groups 3 (XRCC3) participates in DNA double-strand break/recombination repair, and likely also participates in HRR [10].

Several common and putatively functional single nucleotide polymorphisms (SNPs) of ERCC1, XRCC1, and XRCC3 have been identified, of which ERCC1 C118T and ERCC1 C8092A affect ERCC1 mRNA expression, whereas XRCC1 Arg194Trp and XRCC1 Arg399Gln, and XRCC3 Thr241Met are associated with suboptimal DNA repair capacity [9,11], and with an altered risk of several types of cancer [12–15]. Possible associations of polymorphisms in ERCC1 and XRCC1 genes with glioma risk have been examined in European populations with conflicting results [16–19]. However, there have been few reports about the independent and combined roles of ERCC1, XRCC1, and XRCC3 polymorphisms in Chinese populations. Therefore, we conducted a large case-control study to investigate whether there is an association between glioma risk and polymorphisms in these three genes, in a Chinese population.

## 2. Results and Discussion

### 2.1. Results

A total of 478 cases were asked to participate, of whom 443 were successfully interviewed, and provided 5 mL blood samples, for a participation rate of 92.7%. The mean ages of the cases, and controls, were 50.9 ± 9.6 years and 51.2 ± 9.1 years, respectively (Table 1). There were no significant differences in the ages or sex distributions between the two groups (*p* > 0.05). However, the glioma patients were more likely to have had a greater exposure history to occupational IR (4.6% *vs.* 1%, *p* < 0.05) and a family history of cancer when compared to the controls (21.4 *vs.* 11.7, *p* < 0.05). Of the cancer cases, 49.5% were astrocytoma.

The minor allele frequencies, among selected controls of ERCC1 C118T, ERCC1 C8092A, XRCC1 Arg194Trp, XRCC1 Arg399Gln and XRCC3 Thr241Met, were consistent with the Minor Allele Frequency (MAF) from the NCBI SNP database (Table 2). ERCC1 C118T, ERCC1 C8092A, XRCC1 Arg194Trp, XRCC1 Arg399Gln, and XRCC3 Thr241Met, were in line with the Hardy-Weinberg equilibrium in controls (all *p* values > 0.05).

The genotype distributions of ERCC1 C8092A and XRCC3 Thr241Met were significantly different between cases and controls (Table 3). Associations between these SNPs, and the risk of glioma, were analyzed using conditional logistical regression analysis, with frequency matched by age and sex. For XRCC1 Arg194Trp, the variant genotype T/T was strongly significantly associated with a lower risk of glioma cancer, when compared with the wild-type C/C, with an adjusted OR (95% CI) of 2.45 (1.43–4.45). The XRCC1 194T allele was associated with elevated susceptibility to glioma (OR = 1.76, 95% CI = 1.21–2.06). Individuals carrying the XRCC1 399A allele had a higher risk of glioma (OR = 1.33, 95% CI = 1.02–1.64), as did those with the XRCC3 241T/T genotype (OR = 3.78, 95%CI = 1.86–9.06). The T allele of XRCC3 Thr241Met was significantly associated with a small increased risk of glioma (OR = 1.38, 95% CI = 1.04–1.65).

A further association analysis was conducted to identify the interactions of two susceptibility-associated SNPs, XRCC1 Arg194Trp, and XRCC3 Thr241Met, and their impact on glioma risk. The combination genotype of the XRCC1 194T allele and the XRCC3 241T allele was associated with higher glioma risk (OR = 2.75, 95% CI = 1.54–4.04) (Table 4).

### 2.2. Discussion

In this case-control study in a Chinese population, we identified the separate and combined effects on the risk of glioma of polymorphisms in ERCC1 C118T, ERCC1 C8092A, XRCC1 Arg194Trp, XRCC1 Arg399Gln, and XRCC3 Thr241Me. We found that XRCC1 194T/T and XRCC3 241T/T were strongly associated with glioma cancer risk, both individually and in combination.

To the best of our knowledge, our study is the first to describe the associations of these DNA repair gene polymorphisms with glioma risk in a Chinese population. Previous studies have focused on only one or two variants in the ERCC1 and XRCC1 genes, which might not sufficiently capture the effect of susceptibility loci in Chinese glioma patients. A recent Brazilian study, with 80 glioma cases and 100 controls, found that XRCC1 194T/T is associated with a strong increased risk of glioma [19]. Another study conducted in southern China, with 127 glioma cases, showed that the homozygous T/T and heterozygotes C/T variants of XRCC1 codon 194, brought a 2.12-fold and 1.46-fold increased risk of glioma when compared to the homozygous wild-type genotype [20]. Our findings strongly indicate that polymorphisms in XRCC1 Arg194Trp and XRCC3 Thr241Met contribute to glioma susceptibility, and are in line with those of previous studies showing that the XRCC1 194T allele is associated with increased glioma risk [19,20]. However, other studies have obtained conflicting results. One hospital-based study, with 271 cases, reported a non-significant association between the XRCC1 194T allele and glioma risk [21], and another large sample study with 701 cases also reported a non-significant increased risk of glioma [18]. The inconsistency of these studies may be explained by differences in genetic origin, population background, source of controls, and sample size, or by chance. Alternatively, gene-environment interactions may operate in the pathogenesis of glioma, and thus differences in environmental risk factors may affect glioma risk.

Our study found a slight increased risk of glioma for patients with XRCC1 399Gln/Gln. Several previous studies have also shown that this allele is a risk factor for glioma [20–23]. In a recent study in a Caucasian population, with 373 glioma patients and 365 controls, there was an increased risk of glioma among patients with the XRCC1 399 A/A genotype [23]. Another study reported that this genotype carried a 3.5-fold risk of glioma in a Turkish population [22]. This solidly positive association from above studies seems to be in line with the well-documented functional relevance of this genotype. As far as we know, the XRCC1 Arg399Gln SNP has been a particular research focus, mainly due to its location within the BRCT1 binding domain [24,25], which interacts with Poly(ADP-ribose)polymerase-1(PARP-1), and thus may result in deficient DNA repair. More recently, Taylor *et al.* showed that the BRCT domain is critical for efficient single-strand break repair and cell survival [11], and that mutations of the BRCT1 domain of BRCA1 may alter the function of the tumor suppressor gene, and thus increase susceptibility to glioma [26]. XRCC1 Arg399Gln expression is reportedly associated with increased gene expression, as measured by mRNA levels in breast cancer patients [12]. Actually, there was no elevated DNA repair activity in the variant cells. The elevated gene expression might be induced by the variant in a structural region of the gene that theoretically influence the enzyme function but not gene expression

We found that patients with homozygous wild-type genotype XRCC3 had a higher risk of glioma than did those with other genotypes. Similar associations have been identified for other types of cancer, including breast cancer, lung cancer, colorectal cancer, skin melanoma, and gastric cancer [14,15,27–30]. One meta-analysis reported that the XRCC3 241T allele is associated with increased risk for breast cancer in Asian and Caucasian populations [30]. Polymorphisms in DNA repair genes may be associated with differences in repair of DNA damage, and thus influence the risk for developing tumors [31]. An association between cancer risk and XRCC3 Thr241Met was also found in glioma patients in previous studies. A study conducted in a Chinese population, with a large sample size, showed that the XRCC3 241T/T genotype may contribute to the development of glioma [23], and a Brazilian study reported a strongly increased risk of glioma among patients with the 241T allele [13]. Our data showed that the XRCC3 241T allele had the similar role in previous studies for glioma risk, suggesting that XRCC3 Thr241Met is involved in susceptibility for developing glioma.

The combination of the XRCC1 194T and XRCC3 241T alleles was also strongly associated with glioma in our study. This combination effect could be explained by the additive effect of the two genotypes. This additive effect of XRCC1 and XRCC3 has also been reported in a western population [18].

## 3. Experimental Section

### 3.1. Study Subjects

This case-control study was conducted at the Shengjing Affiliated Hospital of China Medical University. Between October 2007 and January 2012, all hospital patients with newly diagnosed, histologically confirmed primary gliomas, whose first visit fell within two months of initial diagnosis, were asked to participate in the study. Those who consented were interviewed and provided 5 mL blood samples. Controls were selected from among inpatients from the orthopedics, dermatology, and digestive departments; controls had to lack a prior history of cancer, and were frequency matched to cases by age (within 5 years), and sex.

A self-designed questionnaire was used to collect data on demographics and potential risk factors, including smoking, alcohol consumption, and family history of cancer, as well as occupational infrared ray (IR) exposure history.

The research protocol was approved by the ethics committees of the Shengjing Affiliated Hospital of China Medical University, and informed consent was obtained from all recruited subjects.

### 3.2. Genotyping

DNA was extracted from the buffy-coat fractions with the TIANamp blood DNA kit (Tiangen Biotech, Beijing, China). SNP genotyping was performed in a 384-well plate format on the Sequenom MassARRAY platform (Sequenom, San Diego, CA, USA). Primers for polymerase chain reaction (PCR) amplification and single base extension (SBE) assays were designed by Sequenom Assay Design 3.1 software (Sequenom, San Diego, CA, USA) according to the manufacturer’s instructions (Table 5). The PCR was performed with 5 ng of genomic DNA, in a reaction volume of 5 μL, using GeneAmp^®^ PCR System 9700 with Dual 384-Well Sample Block Module (Applied Biosystems, Carlsbad, CA, USA). The excess dNTPs was removed by shrimp alkaline phosphatase enzyme solution (Sequenom), and iPLEX^®^ Gold SBE chemistry (Sequenom) was used to perform base extension reaction. The final base extension products were treated with CLEAN resin (Sequenom) to remove salts. A total of 10 nL of reaction solution was dispensed onto a 384 format SpectroCHIP microarray (Sequenom). The MassARRAY Analyzer Compact with ACQUAIRE Module (Sequenom) acquired spectra from the SpectroCHIP, and spectral data were automatically processed and saved to the MassARRAY database. For quality control, genotyping was performed without knowledge of the case/control status of the subjects, and a random sample of 5% of cases and controls was genotyped again by different researchers. The reproducibility was 100%. DNA extraction and SNP genotyping were conducted in the Shengjing Affiliated Hospital of China Medical University.

### 3.3. Statistical Analysis

All statistical analyses were performed by Stata 8.0 (StataCorp, College Station, USA) and conducted by Dr. W.R. Pan WR. Continuous variables were expressed as mean ± standard deviation (SD), while categorical variables were shown as frequencies and percentages. Demographic characteristics were compared between cases and controls by means of a chi-square test and Student’s t test. The Hardy-Weinberg equilibrium (HWE) was checked for controls with the chi-square test. Conditional logistic regression was used to calculate the odds ratios (ORs) and 95% confidence intervals (CIs). Because of the low allele frequencies, and relative rarity of the homozygous variant genotypes, we combined the homozygous variant and heterozygous groups for analysis. All comparisons were two-sided, and *p* < 0.05 was regarded as statistically significant.

## 4. Conclusions

In conclusion, we found that the polymorphisms XRCC1 Arg194Trp, XRCC1 Arg399Gln, and XRCC3 Thr241Met, were significantly associated with glioma cancer susceptibility among Chinese women, and that the combination of XRCC1 194T allele and XRCC3 241T allele was even more strongly associated with elevated glioma risk. Our results support the hypothesis that naturally occurring genetic variation in the X-ray repair cross-complementing group of genes increases susceptibility to glioma.

## Figures and Tables

**Table 1:** Characteristics of glioma cases and controls.

Characteristics	Cases *N* = 443	%	Controls *N* = 443	%	χ^2^	*p* value
Age (mean ± SD), years	50.9 ± 9.6		51.2 ± 9.1			
**Age (years)**						
<30	73	16.4	70	15.7	115.2	0.95
30–50	152	34.2	155	35.1		
>50	219	49.4	218	49.2		
**Gender**						
Male	257	58.0	257	58.0	0	1.0
Female	186	42.0	186	42.0		
**Smoking status**						
Smokers	152	34.2	121	27.4	5.09	<0.05
Non-smokers	291	65.8	322	72.6		
**Drinking status**						
Drinkers	212	47.8	197	44.5	1.02	0.31
Non-drinkers	231	52.2	246	55.5		
**Occupational IR exposure history**						
Yes	20	4.6	4	1	3.82	<0.05
No	423	95.4	439	99		
**Family history of cancer**						
Yes	95	21.4	52	11.7	15.08	<0.05
No	348	78.6	391	88.3		
**Histological type**						
Astrocytoma	250	56.5				
Ependymoma	27	6.2				
Glioblastoma	59	13.3				
Oligodendroglioma	11	2.5				
Other	95	21.5				

**Table 2:** Genotype characteristics of the five single nucleotide polymorphisms (SNPs).

Gene name	Single nucleotide polymorphism	Alleles	MAF a			HWE (*p* value) b Control

			Case	Control	From dbSNP	
ERCC1 C118T	rs11615	T/C	0.372	0.341	0.36	0.306
ERCC1 C8092A	rs3212986	G/T	0.292	0.275	0.25	0.133
XRCC1 Arg194Trp	rs1799782	C/T	0.191	0.148	0.13	0.103
XRCC1 Arg399Gln	rs25487	G/A	0.275	0.249	0.26	0.419
XRCC3 Thr241Met	rs861539	T/C	0.287	0.246	0.25	0.092

a Minor Allele Frequency;

b Hardy-Weinberg equilibrium.

**Table 3:** Genotype distributions and association with glioma cancer.

Single nucleotide polymorphism	Cases *N* = 443	%	Controls *N* = 443	*%*	*p* value	OR a (95% CI)	OR b (95% CI)
**ERCC1 C118T (rs11615)**							
T/T	193	43.5	211	47.6	0.45	1.0 (Ref.)	1.0 (Ref.)
C/T	171	38.6	162	36.6		1.15 (0.85–1.56)	1.32 (0.89–1.67)
C/C	79	17.9	70	15.8		1.23 (0.83–1.83)	1.38 (0.91–1.94)
C allele	250	56.5	232	52.4		1.18 (0.89–1.55)	1.27 (0.96–1.67)
**ERCC1 C8092A (rs3212986)**							
G/G	229	51.8	241	54.3	<0.05	1.0 (Ref.)	1.0 (Ref.)
G/T	169	38.1	162	36.5		1.10 (0.82–1.47)	1.24 (0.93–1.61)
T/T	45	10.1	41	9.2		1.15 (0.71–1.88)	1.30 (0.88–1.93)
T allele	214	48.2	202	45.7		1.16 (0.87–1.54)	1.33 (0.98–1.66)
**XRCC1 Arg194Trp (rs1799782)**							
C/C	301	67.9	327	73.8	0.06	1.0 (Ref.)	1.0 (Ref.)
C/T	116	26.1	101	22.9		1.24 (0.91–1.72)	1.36 (0.97–1.83)
T/T	27	6	15	3.3		1.98 (1.01–4.03)	2.45 (1.43–4.45)
T allele	142	42.6	116	34.8		1.33 (0.98–1.80)	1.76 (1.21–2.06)
**XRCC1 Arg399Gln (rs25487)**							
G/G	226	51.1	244	55.1	0.4	1.0 (Ref.)	1.0 (Ref.)
G/A	190	42.8	178	40.1		1.15 (0.87–1.53)	1.28 (0.97–1.66)
A/A	27	6.1	21	4.8		1.39 (0.73–2.66)	1.54 (0.88–2.87)
A allele	217	64.9	199	59.6		1.19 (0.90–1.55)	1.33 (1.02–1.64)
**XRCC3 Thr241Met (rs861539)**							
C/C	217	48.9	234	52.8	<0.05	1.0 (Ref.)	1.0 (Ref.)
C/T	198	44.8	200	45.2		1.07 (0.81–1.41)	1.22 (0.96–1.54)
T/T	28	6.3	9	2		3.35 (1.49–8.25)	3.78 (1.86–9.06)
T allele	226	67.8	209	62.6		1.17 (0.89–1.53)	(1.04–1.65)

a Not adjusted;

b Adjusted for smoking, alcohol drinking, and family history of cancer, as well as occupational infrared ray (IR) exposure history; Ref.: reference.

**Table 4:** Interactions of XRCC1 A194T and XRCC3 C241T in cases and controls on glioma risk.

Single nucleotide polymorphism	Cases *N* = 443	%	Controls *N* = 443	*%*	OR (95% CI)	OR (95% CI)
**XRCC1 Arg194Trp/XRCC3 Thr241Met**						
CC/CC	140	31.6	149	33.6	1.0 (Ref.)	1.0 (Ref.)
T allele/CC	77	17.4	85	19.2	1.02 (0.64–1.44)	1.12 (0.78–1.61)
CC/T allele	161	36.3	178	40.2	0.98 (0.71–1.37)	1.03 (0.83–1.52)
T allele/T allele	65	14.7	31	7.0	2.24 (1.35–3.76)	2.75 (1.54–4.04)

Ref.: reference.

**Table 5:** PCR primers of selected SNPs.

Single nucleotide polymorphism	Primer	Sequence	Extension primer
ERCC1 C118T (rs11615)	1^st^-primer	ACGTTGGATGCTAGACCCTAGCAACTCCAG	AGCAACTCCAGGCTAGAGGGCA
	2^nd^-primer	ACGTTGGATGAATCAGCAGCGCGGACCCCT	

ERCC1 C8092A (rs3212986)	1^st^-primer	ACGTTGGATGGCTCACCTGGTGATGTCTT	CTGGTGATGTCTTGTTGATCC
	2^nd^-primer	ACGTTGGATGTGAAGGAGGAGGATGAGAGC	

XRCC1 Arg194Trp (rs1799782)	1^st^-primer	ACGTTGGATGCCTAGCAACTCCAGGCTAGA	CTGGTGATGTCTTGTTGATCC
	2^nd^-primer	ACGTTGGATGAGCCAATCAGCAGCGCGGAC	

XRCC1 Arg399Gln (rs25487)	1^st^-primer	ACGTTGGATGAGATGCTGGGTGATTGTTGG	GAGTGTGTGGGAGGGGAG
	2^nd^-primer	ACGTTGGATGATCTTTCTGGCCATCTGTCC	

XRCC3 Thr241Met (rs861539)	1^st^-primer	ACGTTGGATGCAACCCTCTGTGAGTGTGTG	GAGTGTGTGGGAGGGGAG
	2^nd^-primer	ACGTTGGATGAGCACCTCAGAGAGTACAAG	
